# Comparison of Patient Satisfaction Between Virtual Visits During the COVID-19 Pandemic and In-person Visits Pre-pandemic

**DOI:** 10.1177/0003489420977766

**Published:** 2020-11-30

**Authors:** Kyohei Itamura, Dennis M. Tang, Thomas S. Higgins, Franklin L. Rimell, Elisa A. Illing, Jonathan Y. Ting, Matthew K. Lee, Arthur Wu

**Affiliations:** 1 Division of Otolaryngology—Head and Neck Surgery, Cedars-Sinai Medical Center, Los Angeles, CA, USA; 2 Department of Otolaryngology—Head and Neck Surgery, Indiana University, Indianapolis, IN, USA; 3 Department of Otolaryngology—Head and Neck Surgery, Louisville University, Louisville, KY, USA

**Keywords:** telehealth, patient satisfaction, patient experience, virtual medicine, telemedicine, COVID-19

## Abstract

**Objective:**

To compare the patient experience of a virtual otolaryngology clinic visit to an in-person visit, especially with its significantly increased implementation during the COVID-19 pandemic.

**Methods:**

Patient satisfaction (PS) metrics from the Clinician and Group Consumer Assessment of Healthcare Providers and Systems survey were queried from March 1, 2020 to May 1, 2020 for telehealth visits and January 1, 2020 to March 1, 2020 for in-person visits. Overlapping and comparable questions were analyzed using Mann-Whitney *U* test, Chi-square test for independence, and Student’s *t*-test.

**Results:**

There were 1284 partial or complete PS surveys from in-person visits and 221 partial or complete virtual PS surveys. There were statistically significantly worse virtual visit evaluations of provider listening, conveyance of information, likelihood to recommend, and overall provider ratings compared to in-person visits.

**Conclusion:**

Telehealth has become the new norm for most healthcare providers in the United States. This study demonstrates some of the initial shortcomings of telehealth in an otolaryngology practice and identifies challenges with interpersonal communication that may need to be addressed as telehealth becomes increasingly prevalent.

**Level of Evidence:**

3.

## Introduction

The Coronavirus (COVID-19) pandemic has caused drastic changes in the practice of medicine including the field of otolaryngology resulting in the rapid adaptation and implementation of telehealth to comply with social distancing guidelines.^1-9^ Prior to the COVID-19 pandemic, the practice of telehealth was shown to be cost-effective, especially in the treatment of chronic diseases like diabetes mellitus and obesity.^10-14^ With the emphasis on remote care during the COVID-19 pandemic, many practices and hospitals produced policy changes to apply telehealth in both medical and surgical fields.^15^

Unfortunately, the field of otolaryngology has many unique challenges when migrating to telehealth, especially given the importance of physical exams as well as the frequent use of endoscopy and microscopy during complete head and neck evaluations.^16^ Recent advancements and promising new technologies such as remote otoscopy or nasopharyngoscopy have the potential to augment remote evaluation of these patients.^16-20^ Given the likelihood that telemedicine will continue to comprise a significant portion of how healthcare is delivered, it is important to determine if the current practices in telehealth are adequately addressing patients’ needs and concerns.^21^

We previously reported initial findings from the first month of shelter at home orders and broad initiation of telehealth, and we reported that patient satisfaction with virtual visits appeared to be lower than in-person visits, especially for metrics of provider-patient communication.^22^ However, we did not have full access to individual questionnaire data, and no statistical analysis could be performed. The goal of this study was to assess the patient experience during virtual visits at a single-institution, multi-provider otolaryngology practice, during the 2-month period when telehealth was used at its peak, and compare the telemedicine experience to traditional in-person clinic visits during the 2-month period prior to the COVID-19 pandemic.

## Methods

Patients seen at Cedars-Sinai Medical Center in Los Angeles, California are routinely administered anonymous patient satisfaction (PS) questionnaires in both the inpatient and outpatient settings for internal quality assurance and practice improvement opportunities. Specifically, National Research Corporation (NRC) is currently used to administer and collect the Clinician and Group Consumer Assessment of Healthcare Providers and Systems (GC CAHPS) survey across outpatient clinics.^23^ This practice has continued during the recent conversion of most outpatient evaluations to a video telehealth medium, though the questions have been slightly altered to suit the different platform. During this time, providers have used Doximity Dialer™^24^ (San Francisco, CA) video conferencing software for the vast majority of visits, while Facetime™^25^ (Cupertino, CA) has been used for a minority of patient visits. Video virtual visits have been extended to 30 to 45 minutes from the normal 15- to 20-minute in-person time slots (depending on provider) to provide enough time to troubleshoot possible technological issues and for potential inefficiencies in video communication. Institutional Review Board (IRB) exemption was granted for this study since no patient records or personal health information was accessed. Patient satisfaction metrics were queried from March 1, 2020 to May 1, 2020 for telehealth visits and January 1, 2020 to March 1, 2020 for in-person visits for the sixteen otolaryngology providers in our practice. All types of ENT visits were evaluated including general otolaryngology and subspecialty pediatric, otologic, laryngologic, rhinologic, and oncologic visits. Different time periods were chosen for the evaluation of each mode of patient visit because during the period when telehealth visits were predominant, in-person visits were rare and vice versa. Survey questions are shown in Tables 1 and 2. Both surveys were provided to the patient via an email and text message Weblink within 2 days after the visit. All surveys were completed within 1 week of the outpatient visit.

**Table 1.** (A) In-Person PS Survey Questions with Responses Categorized as “yes, definitely,” “yes, mostly,” “yes, somewhat,” and “no.”

Yes, definitely, *N* (%)	Yes, somewhat, *N* (%)	No, *N* (%)	Total *N* (%)
Question 1: Did this provider explain things in a way that was easy to understand?			
1207 (94)	65 (5)	12 (1)	1284 (100)
Question 2: Did this provider give you easy to understand information about these health questions or concerns?			
1131 (94)	63 (5)	8 (1)	1202 (100)
Question 3: Did this provider listen carefully to you?			
1215 (95)	51 (4)	12 (1)	1278 (100)
Question 4: Did this provider seem to know the important information about your medical history?			
1105 (88)	121 (10)	35 (2)	1261 (100)
Question 5: Did this provider show respect for what you had to say?			
1200 (96)	39 (3)	14 (1)	1253 (100)
Question 6: Did this provider spend enough time with you?			
1161 (93)	71 (6)	19 (1)	1251 (100)
Question 7: Would you recommend this provider’s office to your family and friends?			
1147 (94)	44 (4)	30 (2)	1221 (100)

**Table:** (B) In-Person PS Survey Questions with Responses on a Scale from 0 to 10.

0, *N* (%)	1, *N* (%)	2, *N* (%)	3, *N* (%)	4, *N* (%)	5, *N* (%)	6, *N* (%)	7, *N* (%)	8, *N* (%)	9, *N* (%)	10, *N* (%)	Total *N*, %
Question 8: Using any number from 0 to 10, where 0 is the worst provider possible and 10 is the best, what number would you use to rate this provider?											
0 (0)	8 (0.7)	0 (0)	3 (0.2)	2 (0.2)	10 (0.8)	7 (0.6)	18 (1.5)	63 (5.2)	180 (14.8)	927 (76.1)	1218 (100)

**Table 2.** (A) Virtual PS Survey Questions with Responses Categorized as “yes, definitely,” “yes, mostly,” “yes, somewhat,” and “no.”

Yes, definitely, *N* (%)	Yes, mostly, *N* (%)	Yes, somewhat, *N* (%)	No, *N* (%)	Total *N* (%)
Question 1: Did the care provider give you enough information?				
159 (75)	28 (13)	18 (9)	7 (3)	212 (100)
Question 2: Did the care provider listen carefully to you?				
173 (82)	14 (7)	15 (7)	8 (4)	210 (100)
Question 3: Did the care provider seem to know your medical history?				
128 (66)	33 (17)	21 (11)	13 (7)	195 (100)
Question 4: Did you know what to do if you have more questions afterwards?				
135 (66)	36 (18)	23 (11)	11 (6)	205 (100)
Question 5: Did you trust the care provider?				
171 (82)	17 (8)	15 (7)	5 (2)	208 (100)
Question 6: Was the quality of the video or call good enough?				
148 (69)	40 (19)	15 (7)	12 (6)	215 (100)
Question 7: Was this method of connecting with a care provider easy to use?				
145 (67)	28 (13)	33 (15)	11 (5)	217 (100)
Question 8: Were you able to talk to a care provider in a timely manner?				
147 (67)	17 (8)	45 (2)	12 (5)	221 (100)

**Table:** (B) Virtual PS Survey Questions with Responses on a Scale from 0 to 10.

0, *N* (%)	1, *N* (%)	2, *N* (%)	3, *N* (%)	4, *N* (%)	5, *N* (%)	6, *N* (%)	7, *N* (%)	8, *N* (%)	9, *N* (%)	10, *N* (%)	Total *N* (%)
Question 9: Using any number from 0 to 10, where 0 is not at all likely and 10 is extremely likely, how likely would you be to recommend this facility to your family and friends?											
1 (0.5)	2 (1.0)	1 (0.5)	4 (2.0)	0 (0)	0 (0)	1 (0.5)	4 (2.0)	16 (8.0)	24 (12.0)	147 (73.5)	200 (100)
Question 10: Using any number from 0 to 10, where 0 is the worst provider possible and 10 is the best, what number would you use to rate this provider?											
0 (0)	3 (1.5)	2 (1.0)	0 (0)	2 (1.0)	0 (0)	2 (1.0)	5 (2.5)	16 (7.9)	32 (15.8)	140 (69.3)	202 (100)

### Statistical Analysis

Overlapping and comparable questions include those regarding careful listening (Table 1(A) Q3 vs Table 2(A) Q2), knowledge of the patient’s medical history (Table 1(A) Q4 vs Table 2(A) Q3), information provided to the patient (Table 1(A) Q2 vs Table 2(A) Q1), recommendation (Table 1(A) Q7 vs Table 2(B) Q9), and overall provider rating (Table 1(B) Q8 vs Table 2(B) Q10). Wording for the “informative” category was most divergent between the two surveys (Table 1(A) Q2 vs Table 2(A) Q1), but the authors felt it would still be meaningful to compare them. Student’s *t*-test was used to analyze the overall provider rating, which was ranked from 0 to 10 in both surveys. Statistical significance was set at *P* < .05 for all analyses. Of note, the in-person visit survey response Likert scale (3-point scale) differed slightly from the virtual visits survey response Likert scale (4-point scale, including “yes mostly” as an additional option) in most of the remaining questions (Tables 1 and 2). Also, for the question regarding recommendation, the in-person survey reported responses on a 4-point Likert scale while the virtual survey reported responses on an ordinal scale from 0 to 10. Because of this inconsistency between the 2 surveys, 2 statistical tests, Mann-Whitney *U* test and Chi-squared test for Independence, were performed to compare in-person and virtual PS survey data and to determine if different statistical analyses would yield varied results. For the Mann-Whitney *U* test of overlapping questions related to listening, conveyance of information, and knowledge of past medical history, the virtual visit responses of “yes, mostly” and “yes, somewhat” were grouped together. Specifically, the in-person Likert scale was codified as 0 (“No”), 1 (“Yes, somewhat”), 2 (“Yes, definitely”), and the virtual visit Likert scale was codified as 0 (“No”), 1 (“Yes, somewhat” and “Yes, mostly), and 2 (“Yes, definitely”). To compare the likelihood to recommend question, the Mann-Whitney *U* test was performed with responses for the in-person visits codified as 0 (“No”), 3.33 (“Yes, somewhat”), 6.66 (“Yes, mostly”), and 10 (“Yes, definitely”). The 0 to 10 ordinal scale for the virtual visits was codified according to the numeric score: 0 (“0”), 1 (“1”), . . ., 10 (“10”).

The Chi-squared test was used to dichotomize responses from the 2 surveys in order to compare them. For questions regarding listening, information, and past medical history, “yes, definitely” and “yes, mostly” were grouped together as positive responses and “yes, somewhat” and “no” were grouped together as negative responses. For the question of likelihood to recommend, “yes, definitely,” “yes, mostly” and “10” were grouped together as positive responses and “yes, somewhat”, “no” and “0–9” were grouped together as negative responses. We decided to group “yes, somewhat” with negative responses since a 2 out of 3 (0.66) or 2 out of 4 (0.5) rating has been typically viewed as sub-standard at our institution. Average rating of 0.9 has been used as a cutoff for positive patient response in physician reviews. All analyses were performed on Microsoft Excel (Redmond, WA).

## Results

### Patient Satisfaction Responses from In-person Visits

From January 1, 2020 to March 1, 2020, there were 1284 partial or complete PS surveys from in-person otolaryngology office visits at Cedars-Sinai Medical Center. The response rate was 20%. Table 1(A) shows the group of questions with responses categorized as “yes, definitely,” “yes somewhat,” and “no” and their relative frequencies. Table 1(B) shows the group of questions with responses on a scale from 0 to 10.

### Patient Satisfaction Responses from Virtual Visits

From March 1, 2020 to May 1, 2020, there were 221 partial or complete PS surveys at Cedars-Sinai Medical Center. The response rate was 25%. Survey responses and their relative frequencies are shown in Table 2. Table 2(A) shows the group of questions with responses categorized as “yes, definitely,” “yes, mostly,” “yes somewhat,” and “no.” Table 2(B) shows the group of questions with responses on a scale from 0 to 10.

### Comparison of Overlapping Survey Questions from In-person and Virtual Visits

**Figure 1.**
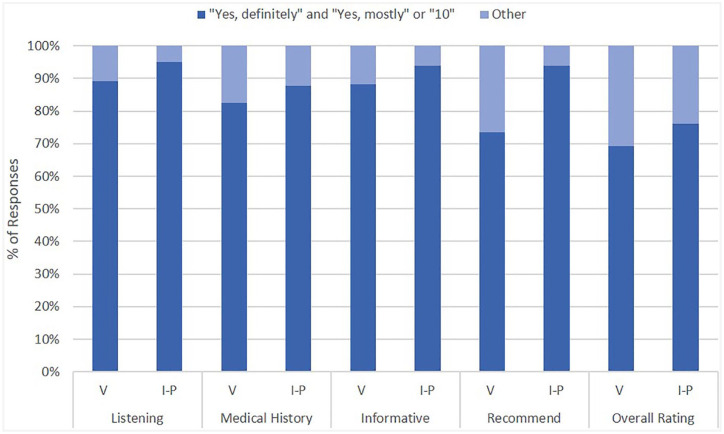
Percentage of ideal scores for overlapping PS survey questions between in-person and virtual office visits. Abbreviations: V, virtual visit; I-P, in-person.

**Figure 2.**
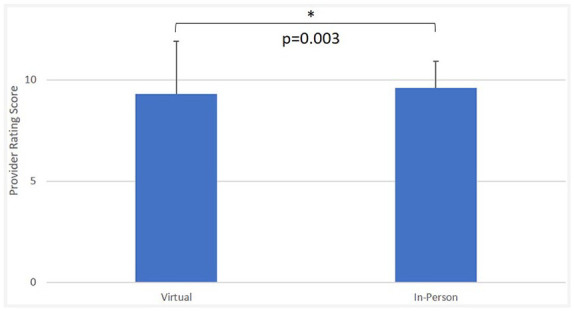
In-person versus virtual office visit overall provider rating. *Note.* T-test results comparing overall provider rating score between virtual and in-person visits.

Survey questions that overlapped between in-person and virtual PS surveys are summarized in Figure 1. In all 5 overlapping questions, there was a lower percentage of responses for virtual visits that reported more satisfactory evaluations of “Yes, definitely” and “Yes, mostly” or “10” compared to those of in-person visits. Student’s *t*-test demonstrated that the lower overall provider rating for virtual visits (9.3 ± 1.6) compared to in-person visits (9.6 ± 1.1) was significant, *P* = .003 (Figure 2).

Percentage of responses with Chi-squared tests yielded significantly higher satisfaction scores for patients of in-person visits compared to those of virtual visits for the questions regarding careful listening (χ^2^ = 12.0, *P* < .001), the patient’s satisfaction with the amount of information offered by the provider (χ^2^ = 97.9, *P* < .001), likelihood to recommend to family and friends (χ^2^ = 88.2, *P* < .001), and overall provider rating (χ^2^ = 4.3, *P* = .04). Though not statistically significant, knowledge of the patient’s medical history by the provider was also associated with higher satisfaction scores (χ^2^ = 3.8, *P* = .05).

**Figure 3.**
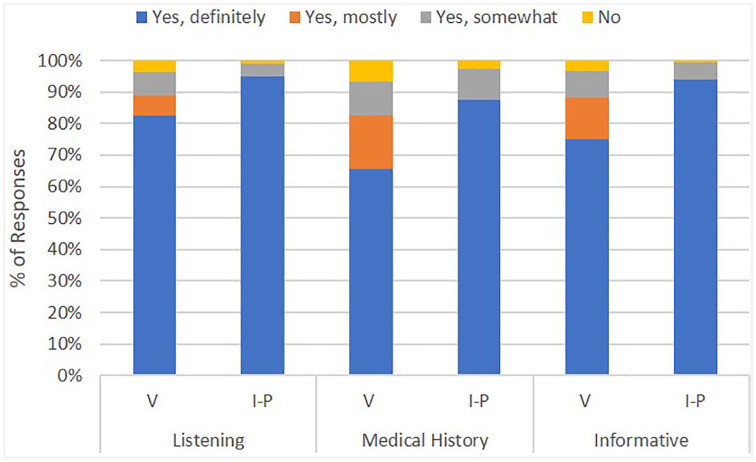
Percentage of responses by Likert-scale for overlapping questions. Abbreviations: V, virtual visit; I-P, in-person.

Mann-Whitney *U* tests yielded significantly higher satisfaction scores for patients of in-person visits compared to those of virtual visits for the questions regarding careful listening by the provider (*U* = 117 132, *P* = .003), knowledge of the patient’s medical history by the provider (*U* = 96 072, *P* < .001), and patient’s satisfaction with the amount of information offered by the provider (*U* = 103 049, *P* < .001) (Figure 3). A higher likelihood to recommend score was observed with patients of in-person office visits (*U* = 98 737, *P* < .001).

Despite the physician allotting double the time per visit, scores for non-overlapping telehealth survey questions were also low in certain categories (Table 2). Significant proportions of patients responded negatively (“yes, somewhat” + “no”) for ease of connection to the provider (44%), video quality (27%), wait times (57%), and patient understanding of what to do for follow-up questions (34%).

## Discussion

The response to prevent the rapid spread of COVID-19 has been reliant on social distancing.^26^ To comply with this, many medical practices have shifted towards virtual visits.^27^ In an effort to encourage the use of telehealth, major revisions have been made by reimbursing entities, such as the Center for Medicare and Medicaid Services, to allow for clinicians to provide more services to patients via telehealth. Despite the widespread implementation of telehealth, there are few studies that critically evaluate the quality of virtual visits.

Evidence-based adoption of telehealth historically has been relatively limited in otolaryngology compared to other specialties owed to the unique challenges of remote examination of the head and neck. These current circumstances, however, serve as an unprecedented catalyst for many to integrate telehealth into their practices on a large scale.^28,29^

As with any new technology, best practices must be established to ensure the safe delivery of quality care via virtual visits.^30^ To this end, it is imperative to evaluate the subjective experience of patients and the impact of the modality of virtual visits on the patient-physician relationship.^21^ Although accurate diagnosis is essential for any medical evaluation, equally important are accurate and clear communication with patients. Previous studies have shown positive reception in patient with virtual visits, however, there have not been any studies that specifically address patients of an otolaryngologic practice.^31^

In this study, we describe survey results from patients of both virtual and in-person clinic visits to multiple otolaryngology providers at an academic tertiary care practice. We report statistically significant lower scores in questions concerning interpersonal communication skills and provider ratings for patients who underwent a virtual visit compared to an in-office visit. Because the answer options for virtual visits differed slightly, except for in the overall provider rating question, we compared overlapping questions using 2 different modes of analysis. Both methods demonstrated significantly lower evaluations of interpersonal communication (listening, conveyance of information). Ratings that evaluated the visit as a whole (i.e., overall provider rating, likelihood to recommend) also demonstrated significantly worse scores for virtual visits. While the absolute difference between the 2 modes might appear minimal (9.3 vs 9.6, respectively for overall provider rating), small differences such as these may be clinically meaningful and not just statistically significant if they are borne out in larger and longer term studies.

Although the reason underlying these observations is speculative at best, we postulate that the patient’s subjective experience was heavily influenced by the telemedicine platform. Patients complained of difficulties in communication and longer wait times despite having double the length of time allocated for a virtual visit compared to an in-office visit. Video quality was frequently rated as low. This may be reflective of several factors that may be difficult to overcome including internet bandwidth and server speed of each platform (Doximity, Apple, etc.). A poor video quality will have cascading effects on the entire encounter. A common source of frustration for all users of video conferencing software is lag which may result in individuals speaking over each other or loss of important information. The internet speed for patients was not evaluated; however, this may be an important variable to consider in the future to optimize the patient experience. The wait time prior to speaking with the provider was also poorly rated. One explanation might be the change in patient intake such that patients are now being checked in virtually by the staff ahead of their appointment times. Completing the past medical history, review of systems, and patient reported outcome measure (PROM) surveys may take longer for patients and staff to accomplish over a video visit. These issues may also have been exacerbated by the need to rapidly adapt these practices without formal training. We would expect that patient satisfaction will increase as we resolve technical difficulties and move past the learning curve.

Although the implementation and adaptation of virtual visit by otolaryngologists have been rapid in response to COVID-19, it is highly likely that virtual visits will continue to play an important role even as COVID-19 precautions are relaxed. Even though there are various advantages in using telemedicine, we observe that there is ample room to improve the patient experience. Further study is warranted to determine how to optimize this mode of clinical care for otolaryngology and to identify potential or inherent pitfalls in this technology.

Various limitations merit discussion. Response rates for the video and in-person visits were 25 and 20%, respectively, which could create a selection bias. However, these response rates are comparable to the overall response rate of 20% that our institution has tracked across all specialties and clinics prior to the pandemic. This study was conducted at a single institute during the initial 2 months of quarantine in the Los Angeles area, and a significant selection bias may exist due to the novelty of telehealth for Otolaryngology providers. This rapid implementation void of extensive training for both providers and patients could skew the results toward the negative experience. Relative dissatisfaction with virtual visits may have also been a manifestation of patient frustration with the platform or inability to be seen in person rather than a judgment of the actual clinical encounter. Additionally, the subtly different response choices for video and in-person visits may have led to an inexact comparison between the 2 modes of consultation. This was most significant in the “informative” category. However, we found that analysis using 2 different statistical methods agreed on all overlapping questions except for knowledge of past medical history. Future work will be important to determine changes in the patient experience as we become more familiar with the use of telehealth and the various platforms mature.

## Conclusion

Due to the COVID-19 crisis, telehealth was quickly employed in the delivery of healthcare in the United States. This study demonstrates various deficits in the use of telehealth in the field of otolaryngology with respect to the patient experience. Despite the advantages and utility of telehealth, particularly under physical distancing guidelines, providers should be aware of and address communication challenges inherent in providing remote care.
